# Association between health related quality of life and progression of chronic kidney disease

**DOI:** 10.1038/s41598-019-56102-w

**Published:** 2019-12-20

**Authors:** Tae Ryom Oh, Hong Sang Choi, Chang Seong Kim, Eun Hui Bae, Yun Kyu Oh, Yong-Soo Kim, Kyu Hun Choi, Soo Wan Kim, Seong Kwon Ma

**Affiliations:** 1 Department of Internal Medicine, Chonnam National University Medical School, Gwangju, Korea; 2 Department of Internal Medicine, Seoul National University College of Medicine, Seoul, Korea; 3 Department of Internal Medicine, The Catholic University of Korea, Seoul St. Mary’s Hospital, Seoul, Korea; 4 Department of Internal Medicine, College of Medicine, Institute of Kidney Disease Research, Yonsei University, Seoul, Korea

**Keywords:** Quality of life, End-stage renal disease

## Abstract

The impact of health-related quality of life (HRQOL) on outcomes remains unclear in chronic kidney disease (CKD) patients despite its importance in socioeconomic aspects and individual health. We aim to identify the relationship between HRQOL and progression of CKD in pre-dialysis patients. A total 1622 patients with CKD were analyzed in the KoreaN cohort Study for Outcomes in patients With Chronic Kidney Disease, a prospective cohort study. CKD progression was defined as one or more of the following: initiation of dialysis or transplantation, a two-fold increase in baseline serum creatinine levels, or a 50% decline in the estimated glomerular filtration rate during the follow-up period. The group with CKD progression had lower scores of HRQOL than the group without CKD progression. A fully adjusted Cox proportional hazard ratio model showed that each low baseline physical and mental component summary score was associated with a higher risk of CKD progression. In Kaplan-Meier survival analysis using propensity score matched data, only low physical component summary scores showed statistical significance with CKD progression. Our study highlights low physical component summary score for an important prognostic factor of CKD progression. Risk-modification interventions for high-risk patients may provide benefits to individuals.

## Introduction

The prevalence of chronic kidney disease (CKD) is increasing globally, which is an important public health issue. The increase in the prevalence of CKD is not only a health problem, including mortality^1^, but it also increases the socioeconomic burden. According to the report of the Korea Centers for Disease Control and Prevention, the prevalence rate of CKD in Korea is 13.7%, as in other countries. The total cost of socioeconomic expenditure of chronic kidney disease in 2011 was estimated at 502.19 billion, which was very high compared to that of other diseases, such as chronic obstructive lung disease and chronic liver disease^2^. According to the Health Insurance Review and Assessment Service, total medical expenses for patients with chronic kidney failure increase by an annual average of 14% or more^3^. In the United States and Australia, as well as in Korea, the medical expenditure of chronic kidney disease is a significant part of the total cost of health care^4,5^.

Health-related quality of life (HRQOL) focuses on the impact of health status on the quality of life. End-stage renal disease (ESRD) patients showed lower HRQOL scores than the healthy population^6,7^. Low HRQOL scores in ESRD patients are related to their medical and socioeconomic status. In the KDOQI guideline, HRQOL is considered an important outcome and its repeated measurements are recommended to indicate quality of care in hemodialysis patients^8^. It is well known that low HRQOL scores in ESRD patients are associated with higher mortality and hospitalization rates^9–12^. Similarly, HRQOL is associated with the malnutrition-inflammation score^13^, mortality^14^, and sleep disorders^15^ in renal transplant patients. However, the relationship between low HRQOL score and adverse outcome is less known in pre-dialysis CKD patients, including mortality, cardiovascular outcome, and CKD progression. Some studies have shown that cardiovascular outcome and mortality are associated with low HRQOL in patients with pre-dialysis CKD, but the relationship between HRQOL and CKD progression is controversial^16–18^. It is important to understand the relationship between HRQOL and renal outcome, not just as a consequence of disease. Because HRQOL is affected by symptoms of CKD, side effects of treatment, and its burden^19^. We analyzed the association between HRQOL and CKD progression to improve our understanding of the impact of HRQOL on renal outcome in patients with pre-dialysis CKD.

## Results

### Participants and characteristics

Among a total of 1622 participants, the median (25^th^ percentile; 75^th^ percentile) follow-up period was 3.4 years (2.1; 5.3), the mean ( ± standard deviation) age was 57.6 ± 12.4 years, 38.5% were female, 33.7% had diabetes mellitus, the mean baseline estimated glomerular filtration rate (eGFR) was 54.3 ± 31.3 ml/min/1.73 m^2^, and the median urinary protein excretion was 0.5 mg/gCr (0.1; 1.5). There was a significant difference in baseline characteristics between control and low PCS groups. Compared to the control group, the low PCS group had a higher age, a higher proportion of women, a higher prevalence of diabetes mellitus, and a lower income, education, and employment rate. Regarding laboratory findings, the low PCS group showed lower hemoglobin levels, higher baseline creatinine levels, and more severe proteinuria. For detailed comparison, see Table 1 (before propensity score matching).

**Table 1:** Clinical characteristics of the subjects by baseline physical component score categories.

	Missing value (%)	Total subjects (n = 1622)	Control (n = 1357)	Low PCS (n = 265)	p-value
Age, years	0 (0)	57.6 ± 12.4	51.7 ± 12.4	57.7 ± 11.3	<0.001
Female, n (%)	0 (0)	625 (38.5)	472 (34.8)	153 (57.7)	<0.001
Systolic blood pressure, mmHg	2 (0.1)	127.7 ± 16.1	127.2 ± 15.6	130.6 ± 17.8	0.004
Diastolic blood pressure, mmHg	2 (0.1)	77.2 ± 10.9	77.3 ± 10.9	77.1 ± 11.1	0.785
Mean blood pressure, mmHg	2 (0.1)	93.9 ± 11.4	93.9 ± 11.4	94.9 ± 12.2	0.193
Diabetes mellitus, n (%)	0 (0)	546 (33.7)	420 (31.0)	126 (47.5)	<0.001
Body mass index	8 (0.5)	24.5 ± 3.4	24.5 ± 3.4	24.8 ± 3.5	0.203
Current smoker, n (%)	0 (0)	266 (16.4)	228 (16.8)	38 (14.3%)	0.368
Low income, n (%)	51 (3.1)	352 (22.4)	223 (17.7)	119 (47.0)	<0.001
Low education, n (%)	0 (0)	345 (21.3)	232 (17.1)	113 (42.6)	<0.001
Employed, n (%)	0 (0)	945 (58.3)	864 (63.7)	81 (30.6)	<0.001
**Laboratory findings**					
Hemoglobin, g/dL	0 (0)	12.9 ± 2.0	13.1 ± 1.9	11.7 ± 1.9	<0.001
Creatinine, mg/dL	0 (0)	1.5 [1.0; 2.2]	1.4 [1.0; 2.1]	1.8 [1.3; 2.7]	<0.001
CKD-EPI creatinine equation, ml/min/1.73 m^2^	0 (0)	54.3 ± 31.3	57.0 ± 31.4	40.4 ± 26.8	<0.001
Albumin, g/dL	2 (0.1)	4.2 [4.0; 4.5]	4.3 [4.0; 4.5]	4.2 [3.8; 4.4]	<0.001
Total cholesterol, mg/dL	2 (0.1)	171.0 [147.0; 198.0]	171.0 [147.0; 197.0]	173.0 [146.0; 206.0]	0.309
Low-density lipid (mg/dL)	14 (0.9)	94.0 [74.0; 116.0]	94.0 [74.0; 115.0]	93.5 [72.0; 119.0]	0.935
High-density lipid (mg/dL)	15 (0.9)	47.0 [39.0; 58.0]	47.0 [39.0; 58.0]	47.0 [39.0; 58.0]	0.549
Triglyceride (mg/dL)	32 (2.0)	132.0 [93.0; 193.0]	132.0 [92.0; 190.0]	133.0 [96.0; 200.0]	0.398
hsCRP (mg/dL)	0 (0)	0.6 [0.2; 1.6]	0.6 [0.2; 1.5]	0.8 [0.3; 2.1]	0.001
UPCR (mg/gCr)	35 (2.2)	0.5 [0.1; 1.5]	0.4 [0.1; 1.3]	1.0 [0.3; 2.8]	<0.001

Abbreviations: CKD-EPI, CKD Epidemiology Collaboration; hsCRP, high sensitivity C-reactive protein; UPCR, urine protein–creatinine ratio.

### Association between health-related quality of life and CKD progression

**Figure 1:**
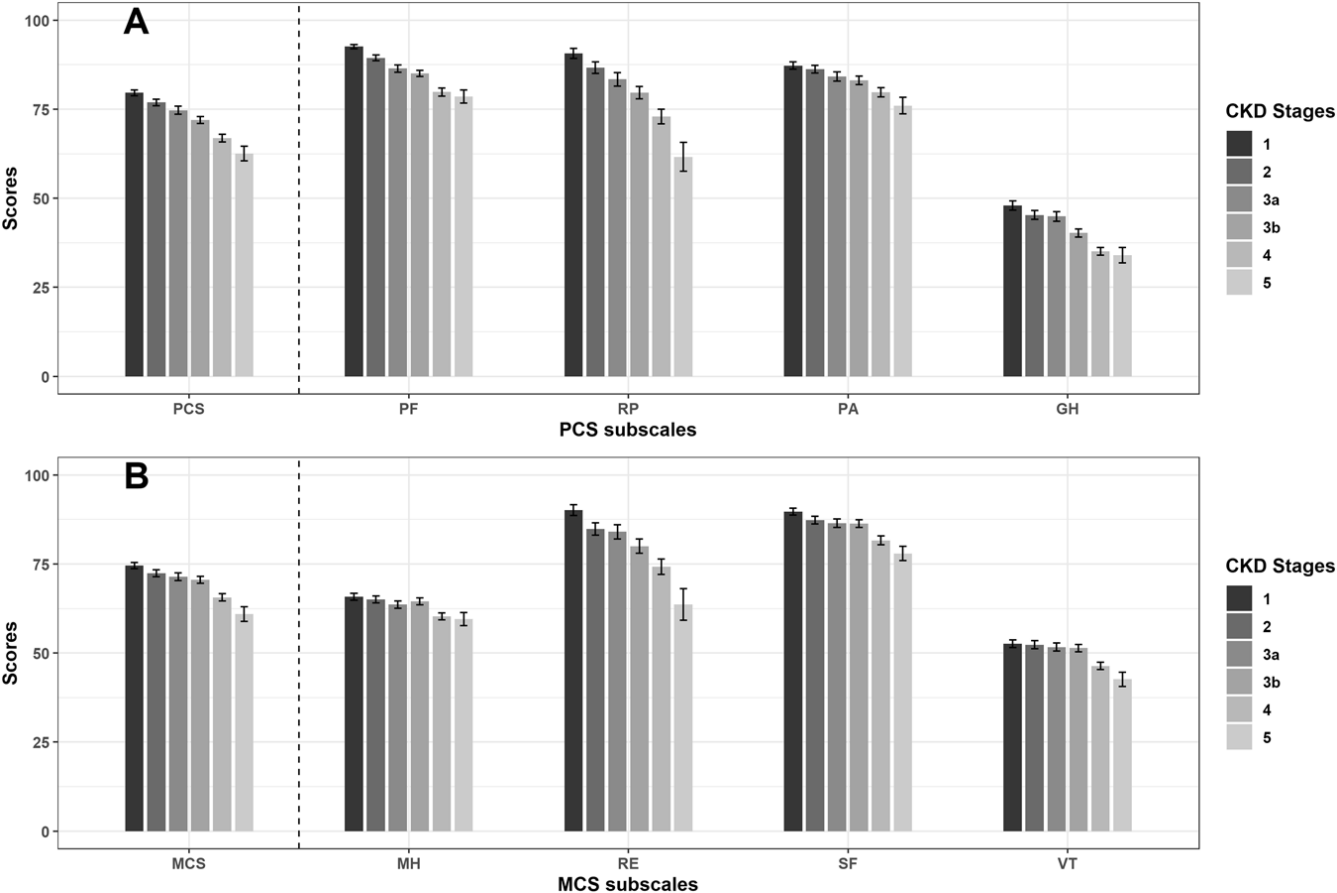
Distribution of the HRQOL scores according to CKD stages. (**A**) Scores for physical component summary and related subscales. (**B**) Scores for mental component summary and related subscales. Abbreviation: CKD, chronic kidney disease; HRQOL, health-related quality of life.

**Figure 2:**
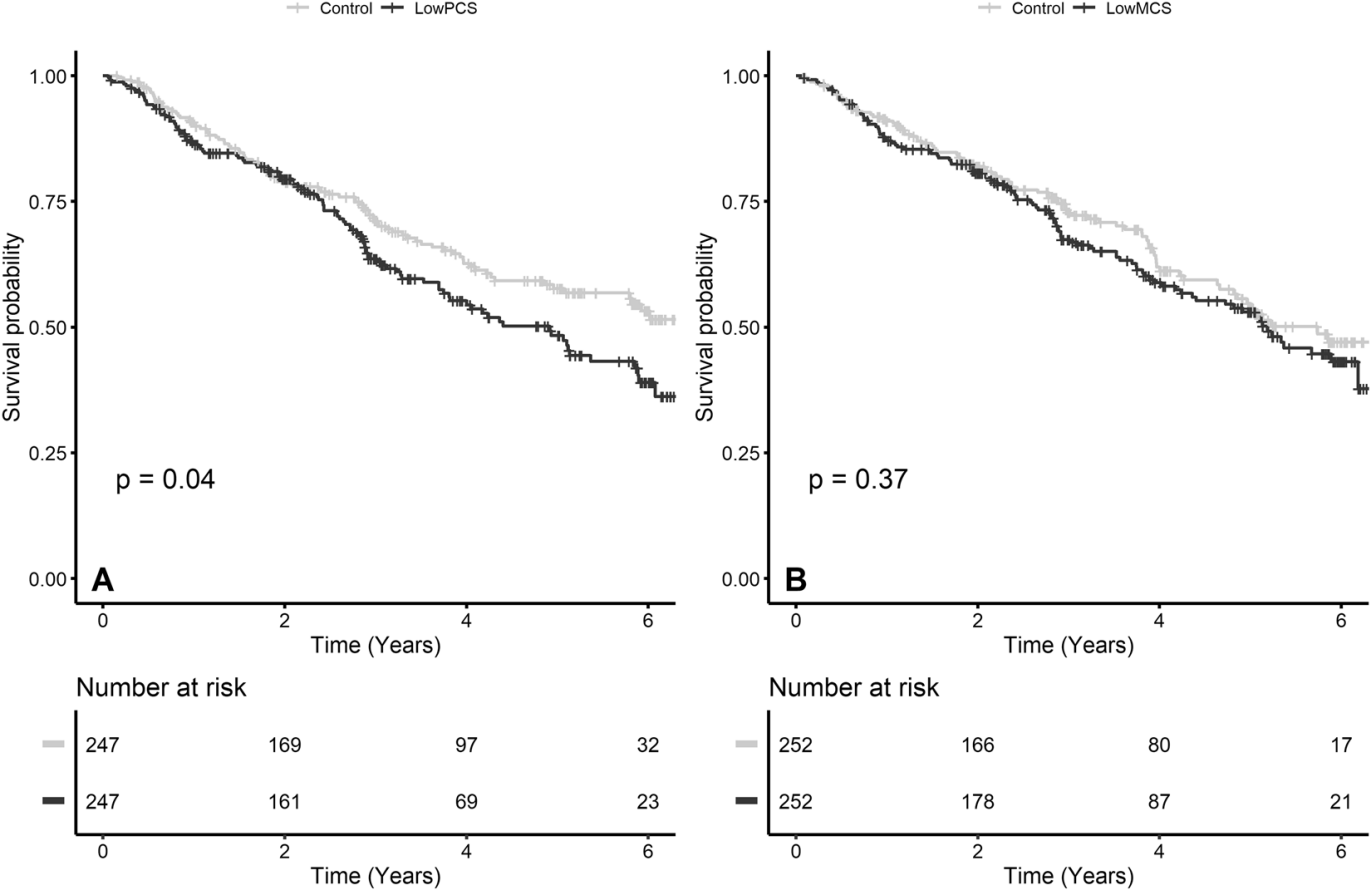
Restricted cubic spline curve for the Cox proportional hazard model of HRQOL. (**A**) A linear relationship between the renal outcome and PCS score was observed. (**B**) A non-linear relationship between the renal outcome and MCS score was observed. Abbreviation: HRQOL, health-related quality of life; MCS, mental component summary; PCS, physical component summary.

CKD progression occurred in 441 patients during the follow-up period. It occurred in 112 (42.3%) patients in the low PCS group and in 329 (24.2%) individuals in the control group. The mean PCS scores of the two groups were 75.5 ± 17.0 for the group without CKD progression and 66.8 ± 19.6 for the group with CKD progression (p < 0.001). In all domains in PCS, the group with CKD progression had lower scores (Supplementary Fig. S1). As with PCS, the groups with or without CKD progression had lower MCS (mental component summary) scores (Supplementary Fig. S2). Additionally, we found that the HRQOL scores decreased with CKD progression (Fig. 1). We assessed the continuous association between HRQOL and renal outcome using a restricted cubic spline curve (Fig. 2). A relatively linear relationship was observed between renal outcomes and PCS compared to MCS. Additionally, both low PCS and MCS were risk factors for CKD progression in the univariate Cox proportional hazard model. In models 2 and 3 of Cox proportional hazard model analysis, albumin violated the proportional hazard assumption and stratified analysis with hypoalbuminemia (No *vs* Yes) was performed. In stratified analysis with model 3, the results of the interaction analyses between hypoalbuminemia and low PCS and between hypoalbuminemia and low MCS were not statistically significant (p for interaction, 0.344 and 0.228, respectively). Low PCS showed a consistent relationship with CKD progression in model 3 as in other models. The hazard ratio (HR) of low PCS in Model 3 was 1.658. This was similar to proteinuria (HR (95% confidence interval (CI)), 1.444 (1.378–1.513); p < 0.001) and baseline creatinine (HR (95% CI), 2.79 (2.565–3.035); p < 0.001) in model 3. In the adjusted Cox proportional hazard model, the effect of low MCS on CKD progression decreased significantly (crude model: HR (95% CI), 2.040 (1.646–2.529); model 1: HR (95% CI), 2.008 (1.616–2.496); model 2: HR (95% CI), 1.228 (0.918–1.644); and model 3: HR (95% CI), 1.291 (1.001–1.665)). Regardless of whether the first 2 years of follow-up were included, we found that statistically significant association between low PCS and CKD progression, but low MCS showed a relatively weak relationship (Table 2). We also analyzed the Cox proportional hazard model with quartiles of the HRQOL scores for sensitivity analysis, which yielded consistent and robust results (Table 2). We also analyzed all subscales of SF-36 separately (Table 3). All items except bodily pain in the PCS subscales showed a statistically significant association with the renal outcome. In the MCS subscales, only vitality showed a significant effect in the fully adjusted model. This is similar to the results of the analysis using component summary scores (Table 2).

**Table 2:** Cox proportional hazard ratio model for HRQOL and composite renal outcome.

Model	Hazard ratio for low PCS (95% confidence interval)		Hazard ratio for low MCS (95% confidence interval)	
	Overall (n = 1622)	2 years lag (n = 1242)	Overall (n = 1622)	2 years lag (n = 1242)
Crude	2.361 (1.904–2.927)	2.213 (1.657–2.956)	2.040 (1.646–2.529)	1.900 (1.424–2.523)
Model 1	2.386 (1.903–2.992)	2.170 (1.603–2.937)	2.008 (1.616–2.496)	1.851 (1.388–2.470)
Model 2	1.702 (1.209–2.027)	1.685 (1.200–2.364)	1.228 (0.918–1.644)	1.448 (1.044–2.008)
Model 3	1.658 (1.300–2.228)	1.936 (1.400–2.678)	1.291 (1.001–1.665)	1.400 (1.019–1.921)
Quartile 1	1 (reference)	1 (reference)	1 (reference)	1 (reference)
Quartile 2	0.770 (0.594–0.998)	0.695 (0.498–0.969)	0.976 (0.754–1.265)	0.808 (0.575–1.136)
Quartile 3	0.711 (0.539–0.941)	0.673 (0.466–0.972)	0.674 (0.507–0.898)	0.679 (0.479–0.961)
Quartile 4	0.454 (0.326–0.632)	0.466 (0.312–0.696)	0.711 (0.532–0.950)	0.682 (0.474–0.981)

In overall follow-up period, model 1 (n = 1622) is adjusted for age and sex. Model 2 and model 3 are stratified with hypoalbuminemia due to violation of proportional hazard assumption. Model 2 (n = 1559) is adjusted for model 1 variables plus mean blood pressure, body mass index, low education, employment, low income, nutritional factors (high sensitivity c-reactive protein and total cholesterol) and presence of severe anemia. Model 3 (n = 1527) is adjusted for model 2 variables plus renal factors (urine protein creatinine ratio and baseline creatinine). The Cox proportional hazard ratio model for quartiles of the HRQOL score was adjusted using the same parameters as those used in model 3.

Abbreviations: MCS, mental component score; PCS, physical component score; HRQOL, health-related quality of life.

**Table 3:** Cox proportional hazard ratio models for the subscales of HRQOL and composite renal outcome.

	Crude		Model 1		Model 2		Model 3	
	HR (95% CI)	p-value	HR (95% CI)	p-value	HR (95% CI)	p-value	HR (95% CI)	p-value
Low PF	2.123 (1.678–2.686)	<0.001	2.096 (1.624–2.703)	<0.001	1.356 (1.027–1.789)	0.032	1.589 (1.206–2.093)	0.001
Low RP	2.420 (1.934–3.028)	<0.001	2.394 (1.902–3.013)	<0.001	1.320 (1.016–1.715)	0.038	1.804 (1.393–2.336)	<0.001
Low PA	1.723 (1.376–2.157)	<0.001	1.703 (1.351–2.146)	<0.001	1.185 (0.917–1.530)	0.194	—	—
Low GH	2.220 (1.812–2.719)	<0.001	2.228 (1.816–2.734)	<0.001	1.620 (1.308–2.007)	<0.001	1.258 (1.005–1.574)	0.045
Low RE	1.902 (1.535–2.355)	<0.001	1.862 (1.496–2.318)	<0.001	1.090 (0.854–1.391)	0.490	—	—
Low MH	1.539 (1.200–1.973)	<0.001	1.559 (1.215–2.002)	<0.001	1.107 (0.849–1.444)	0.452	—	—
Low SF	1.768 (1,419–2.203)	<0.001	1.765 (1.417–2.220)	<0.001	1.288 (1.015–1.633)	0.037	1.180 (0.928–1.501)	0.177
Low VT	1.724 (1.380–2.153)	<0.001	1.769 (1.413–2.214)	<0.001	1.439 (1.135–1.824)	0.003	1.296 (1.013–1.659)	0.039

Model 1 (n = 1622) is adjusted for age and sex. Model 2 and model 3 are stratified with hypoalbuminemia due to violation of proportional hazard assumption. Model 2 (n = 1559) is adjusted for model 1 variables plus mean blood pressure, body mass index, low education, employment, low income, nutritional factors (high sensitivity c-reactive protein and total cholesterol) and presence of severe anemia. Model 3 (n = 1527) is adjusted for model 2 variables plus renal factors (urine protein creatinine ratio and baseline creatinine).

Abbreviation: PF, Physical function; RP, Role physical limitation due to physical problems; PA, Bodily pain; GH, General health; RE, Role-emotional; SF, Social function; VT, Vitality; HRQOL, health-related quality of life; HR, Hazard ratio; CI, Confidence interval.

### Propensity score matching and subgroup analysis

**Figure 3:**
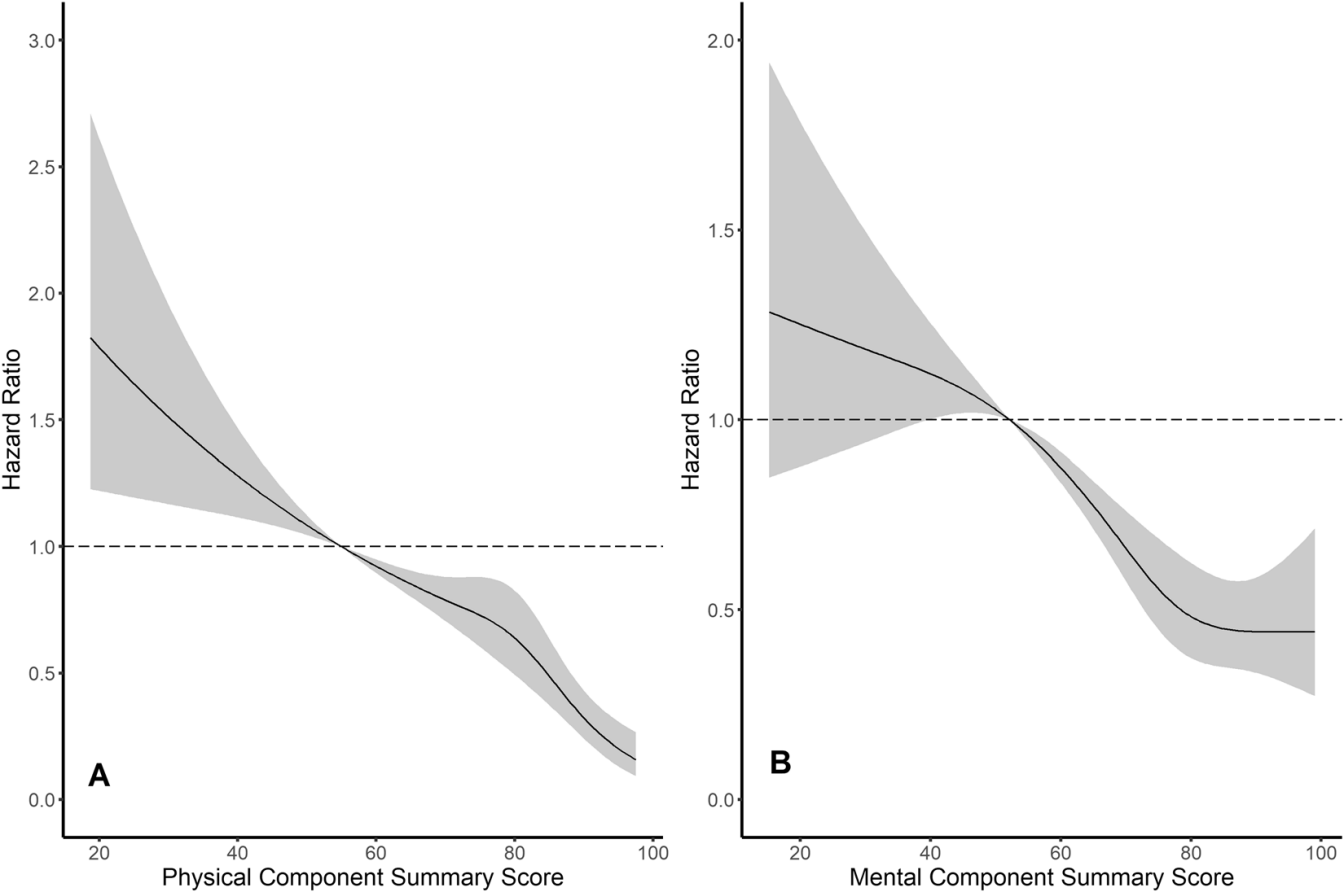
Difference in survival probability between control and low HRQOL group with propensity score matching. (**A**) Kaplan-Meier survival curve for low PCS group. (**B**) Kaplan-Meier survival curve for low MCS group. Abbreviation: HRQOL, health-related quality of life; MCS, mental component summary; PCS, physical component summary.

**Figure 4:**
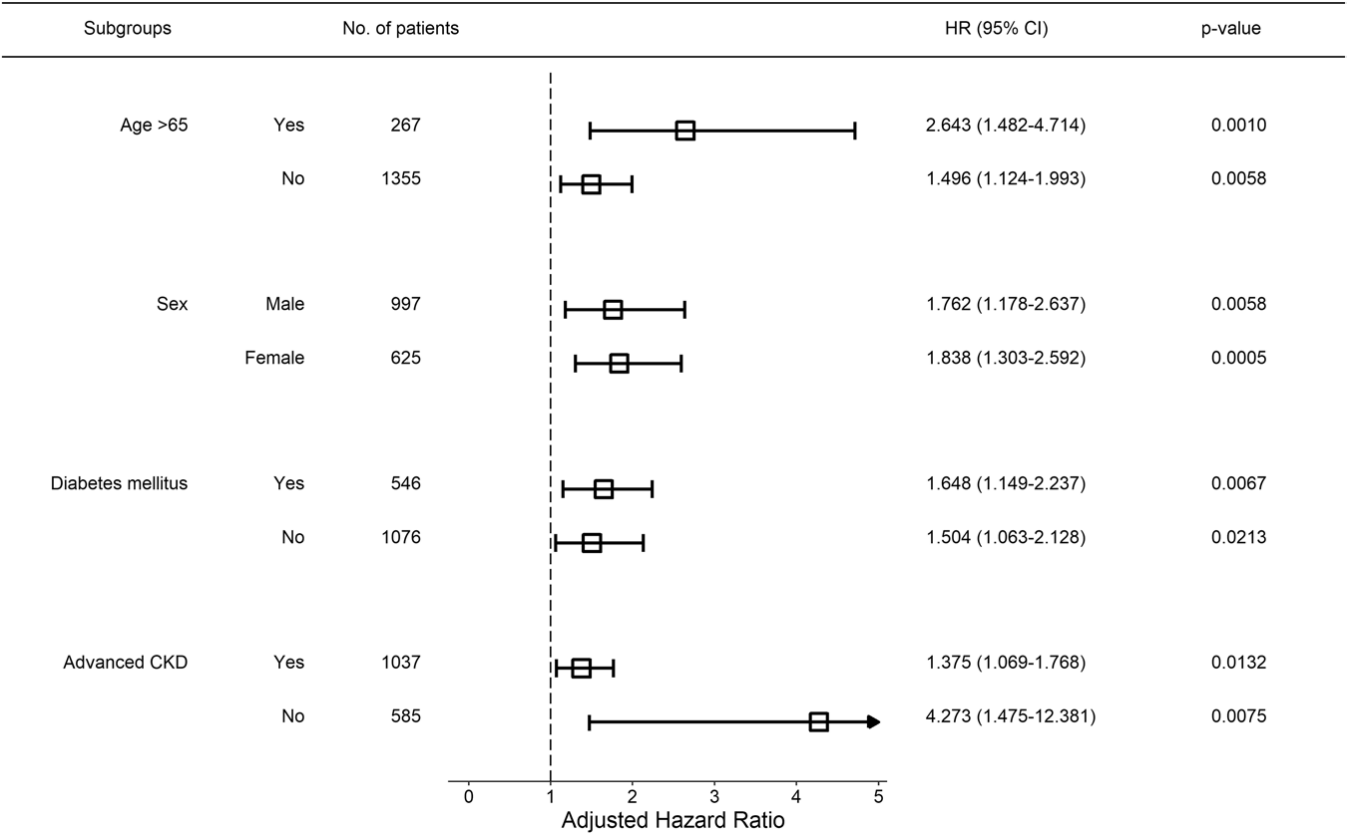
Subgroup analysis for the association between low PCS and CKD progression. Low PCS was a consistent risk factor for CKD progression in all subgroups. Abbreviation: CKD, chronic kidney disease; HRQOL, health-related quality of life; PCS, physical component summary.

As shown in Table 1, there were significant differences in baseline characteristics between the low PCS and control groups. The statistically significant results in Table 2 may have resulted from differences in baseline characteristics. We conducted propensity score matching analysis to control these differences. It is difficult to expect proper matching if too many variables are included in propensity score matching. Therefore, it was performed by selecting the factors that have a great influence on HRQOL. Selected variables included age, sex, employment status, income, education level, nutritional factor (high-sensitivity C-reactive protein and albumin), diabetes mellitus, severe anemia, basal serum creatinine, and proteinuria. Table 4 shows that proper 1:1 matching was achieved. Kaplan–Meier survival analysis was performed using matched data (Fig. 3), and there was no difference in the first two years of follow-up between the two groups, but after two years, there was a significant difference (p = 0.040). Low PCS was an independent risk factor for CKD progression both before and after propensity matching analysis. Additionally, optimal 1:1 propensity score matching was performed for low MCS. Before propensity score matching, low MCS was assumed to be a weak risk factor for CKD progression; however, after propensity score matching, it was found that low MCS was not a risk factor for CKD progression (Fig. 3). Subgroup analysis was conducted by dividing the patients into two groups based on each of the following factors: age ( > 65 years or < 65 years), sex, presence or absence of diabetes mellitus, and eGFR ( < 60 ml/min/1.73 m^2^ or > 60 ml/min/1.73 m^2^); Cox analysis was performed for low PCS in each of the groups. All subgroups showed robust and consistent results. The results are summarized in Fig. 4.

**Table 4:** Results of propensity score matching between control and low PCS groups.

Variables	Before matching				After matching			
	Control (n = 1357)	Low PCS (n = 265)	P-value	Standardized difference	Control (n = 247)	Low PCS (n = 247)	P-value	Standardized difference
Age, year-old	51.7 ± 12.4	57.7 ± 11.3	<0.001	0.506	57.9 ± 11.4	57.5 ± 11.4	0.579	0.017
Female, n (%)	472 (34.8)	153 (57.7)	<0.001	0.473	143 (57.9)	145 (58.7)	0.716	0.016
Severe anemia, n (%)	66 (4.9)	50 (18.9)	<0.001	0.444	40 (16.2)	48 (19.4)	0.560	0.085
Diabetes, n (%)	420 (31.0)	126 (47.5)	<0.001	0.345	116 (47.0)	115 (46.6)	1.000	0.008
Employed, n (%)	864 (63.7)	81 (30.6)	<0.001	0.703	76 (30.8)	74 (30.0)	0.922	0.018
Low income, n (%)	233 (17.7)	119 (47.0)	<0.001	0.661	111 (44.9)	117 (47.4)	0.652	0.049
Low education, n (%)	232 (17.1)	113 (42.6)	<0.001	0.581	97 (39.3)	106 (42.9)	0.464	0.074
C-reactive protein, mg/dL	0.6 [0.2; 1.5]	0.8 [0.3; 2.1]	0.001	0.185	0.7 [0.3; 1.9]	0.8 [0.3; 2.1]	0.611	0.075
Albumin, g/dL	4.3 [4.0; 4.5]	4.2 [3.8; 4.4]	<0.001	0.358	4.1 [3.9; 4.3]	4.2 [3.8; 4.4]	0.426	0.020
Creatinine, g/dL	1.4 [1.0; 2.1]	1.8 [1.3; 2.7]	<0.001	0.367	1.7 [1.2; 3.0]	1.9 [1.3; 2.8]	0.570	0.004
UPCR, mg/gCr	0.4 [0.1; 1.3]	1.0 [0.3; 2.8]	<0.001	0.412	0.9 [0.3; 2.0]	0.9 [0.2; 2.7]	0.631	0.050

Abbreviations: CI, confidence interval; HR, hazard ratio; PCS, physical component score; UPCR, urine protein creatinine ratio.

## Discussion

We found that a lower PCS score significantly increased the risk of CKD progression both before and after propensity score matching. As is well known, HRQOL is a multi-dimensional measurement that is affected not only by the medical condition but also by various sociodemographic factors. In this study, low PCS was associated with several demographic factors including age, sex, low education, low income, presence of diabetes, unemployment, anemia, and decreased renal function. Among the subscales of SF-36, physical function, role of physical limitation, general health, and vitality showed a statistically significant correlation with the renal outcome. Propensity score matching was performed with these parameters that could affect the HRQOL to minimize the effect of confounders, and the sensitivity analysis also showed robust and consistent results.

So far, HRQOL has simply been understood as a result associated with an individual’s illness. HRQOL, however, is becoming important as patient-centered measurements, and it is recognized as an indicator of the health system^20^. SF-36, one of the methods used to measure HRQOL in this study, can be used for clinical research, health policy evaluation, and general population surveys. It deals with the overall aspects of health, including physical and mental conditions. Several studies have confirmed the validity and reliability of SF-36 in obtaining various clinical outcomes and have proven its efficiency in large-scale population studies^21–24^. It is also appropriate to measure the health status of relatively homogeneous group members^25^. Thus, a more sophisticated assessment of HRQOL, measured using SF-36 in CKD patients, may provide insight into treatment decisions between patients and nephrologists and may play an important role in policymaking.

There are a few studies on the association between HRQOL and prognosis in response to these changes, but most of them are limited to ESRD patients, and fewer studies have been conducted on CKD patients. Finding a modifiable risk factor for disease progression in patients with CKD is important not only for individual health but also for socioeconomic reasons. Nevertheless, studies were primarily focused on non-renal outcomes such as mortality and hospitalization. The relationship between CKD progression and HRQOL is still controversial. In a Taiwanese study of CKD patients, low HRQOL was associated with an increased risk of ESRD and death^18^. In the African American Study of Kidney Disease and Hypertension (AASK), low baseline physical HRQOL was associated with a higher risk of cardiovascular events, CKD progression, and death^17^. In Chronic Renal Insufficiency Cohort and Hispanic Chronic Renal Insufficiency Cohort (CRIC and H-CRIC) studies, low HRQOL in patients with CKD showed increased mortality and CV risk but not CKD progression^16^. Although these studies provided important insight on the association between HRQOL and adverse outcomes, it was limited in scope because the Taiwanese study was a small-population study and the AASK study did not consider diabetes mellitus and non-black patients. In CRIC and H-CRIC studies, the influence of the demographic factor was not considered sufficiently in the analysis. The strength of our study is that we appropriately adjusted factors that may affect CKD and HRQOL. In the propensity score matching and sensitivity analyses, we showed robust and consistent results even though we calibrated the interference of various confounding factors as much as possible. However, a recent study has reported that component summary scores are not fully representative of their subscales^26^. In this study, the analysis of each subscale yielded results similar to those of the analysis using component summary scores. Until now, there is no validated instrumental tool to replace component summary scores currently. If a new validated instrumental tool was developed that could be more accurately reflect each subscale, it would be possible to improve the predictive power. We speculate that the exact mechanism of the association of HRQOL and CKD progression is very complicated and difficult. Low PCS may be associated with treatment adherence, which means that self-care is not adequately performed, which may be a risk factor for poor outcomes^27^. In other word, poor physical health can be a surrogate variable which indicate an increase in the burden of disease that patients feel, and it can also be associated with poor prognosis.

The KoreaN Cohort Study for Outcome in Patients With Chronic Kidney Disease (KNOW-CKD) study is a prospective cohort study that enables accurate data collection and management, which increased the reliability of the data. First, as with the limitations of all observative studies, it is impossible to infer certain causality between HRQOL and CKD progression from in this study. Despite the above limitations, observational study is a useful tool for evaluating epidemiological association, so we analyzed the relationship between HRQOL and renal outcome through statistical techniques^28^. Second, the KDQOL-SF36 was originally designed as an instrument for dialysis patients. It has not yet been fully validated for the pre-dialysis CKD patients, although studies are currently on pre-dialysis CKD patients are ongoing^29,30^. Therefore, careful interpretation of the results and further research is required^31^. Third, this study cannot solve the problems of hidden bias and confounding factors, despite the extensive risk adjustment. Fourth, there is no consensus on the clinically meaningful cut-off score of SF-36. This is a limiting factor in the implementation of HQROL in clinical care. It would be more useful if the reference score of HRQOL was defined based on more research.

In conclusion, HRQOL is a significant independent risk factor for CKD progression. It is able to identify a group of patients at high risk of CKD progression by measuring HRQOL in patients with CKD. Identifying high-risk patients may provide preventive benefits to individuals in terms of their health and socioeconomic aspects. Nephrologists need to focus on patients with impaired HRQOL and consider risk-modification intervention. Further researches are needed to clarify the relationship between low HRQOL and renal outcomes. It may help to improve HRQOL and reduce poor prognosis.

## Methods

### Study population and ethics statement

We reviewed baseline data from the KNOW-CKD, a nationwide multicenter prospective cohort study that included non-dialysis patients with stage 1–5 CKD, aged 20–75 years. The detailed design and methods of the KNOW-CKD study have been previously published (NCT01630486 at http://www.clinicaltrials.gov)^32^. All procedures performed in the participants were in accordance with the ethical standards of the institutional and national research committee at which the studies were conducted and with the 1964 Helsinki declaration and its later amendments or comparable ethical standards. The study protocol was approved by the institutional review board at each participating clinical center, as follows: Seoul National University Hospital (1104–089–359), Seoul National University Bundang Hospital (B-1106/129-008), Yonsei University Severance Hospital (4-2011-0163), Kangbuk Samsung Medical Center (2011-01-076), Seoul St. Mary’s Hospital (KC11OIMI0441), Gil Hospital (GIRBA2553), Eulji General Hospital (201105-01), Chonnam National University Hospital (CNUH-2011-092), and Busan Paik Hospital (11-091) in 2011^33^. Written informed consent was obtained from all study participants. The KNOW-CKD study included 2238 patients and this study comprised 1662 patients (Supplementary Fig. S3).

### Data collection and survey instruments

The utilized data in present study was searched using an electronic data management system (PhactaX, Seoul, Republic of Korea) with the help of the data management department of Seoul National University Medical Research Collaborative Center^33^. The items used in this study were as follows: age, sex, comorbid diseases, work status, income, education level, blood pressure, body mass index and initial laboratory measurements including hemoglobin, albumin, creatinine, total cholesterol, triglyceride, high-density lipoprotein cholesterol (HDL-C), low-density lipoprotein cholesterol (LDL-C), high-sensitivity C-reactive protein (hs-CRP), and urinary protein-to-creatinine ratio. We collected venous blood samples after an overnight fast and measured spot urinary metrics such as protein and creatinine with first-voided urine. The traceable isotope-dilution mass spectrometry method was used to measure serum creatinine. All samples collected before May 1, 2013 were retrospectively analyzed using the samples stored in the Biobank and from May 1, 2013, samples were collected from each institution, transferred to the central laboratory, and measured in real time^33^.

### Definition

The Korean version 1.3 of Kidney Disease Quality of Life short form (KDQOL-SF) was used to evaluate HRQOL. The KDQOL-SF contains the Medical Outcome Study Short Form-36 Health Survey (SF-36) which is composed of a physical component summary (PCS) and a mental component summary (MCS). The PCS and MCS each contain four subscales: (1) Physical Function, (2) Role Physical limitation due to physical problems, (3) Bodily Pain, (4) General Health, (5) Vitality, (6) Role-Emotional, (7) Social Function, and (8) Mental Health. The first four subscales comprise the PCS, while the remaining subscales constitute the MCS. The patient’s answers to the survey were translated into SF-36 scores, and each scale was represented by a score from 0 to 100. It means that the higher the score on that scale, the better the HRQOL the patient feels. PCS and MCS scores were the explanatory variables in this study and low HRQOL (low PCS and MCS) was defined as lower than ‘the mean HRQOL score - 1 standard deviation of HRQOL score’ of the entire patient. Participants were divided into a control group and a low HRQOL group based on the scores. In this study, severe anemia is defined as hemoglobin level below 10 g/dL because patients with chronic kidney disease who do not undergo dialysis consider using an erythropoiesis-stimulating agent when hemoglobin levels are less than 10 g/dL, regardless of the sex of the patient. Low-income status was defined as having a monthly family income of ₩1,500,000 or less (approximately United States $1500). Low education level was defined as an academic background of less than high school graduation because high school is a compulsory educational curriculum in Korea. Diabetes mellitus was defined as serum hemoglobin A1c ≥ 6.5%, fasting glucose ≥ 126 mg/dl, or a previous diagnosis of diabetes. The estimated glomerular filtration rate was calculated using the Chronic Kidney Disease Epidemiology Collaboration (CKD-EPI) equation^34^. CKD progression was defined as one or more of the following: initiation of dialysis or kidney transplantation, a two-fold increase in baseline serum creatinine, or a 50% decline in estimated glomerular filtration rate (eGFR) during the follow-up period. The acute effect is defined as an early change in GFR different in direction or magnitude to the changes noted later in GFR, and doubling of creatinine levels corresponds to a 50–57% decrease in eGFR, which showed robust results in a moderate acute effect^35^. Therefore, both doubling of creatinine levels and a 50% decrease in eGFR were considered markers of CKD progression.

### Statistical analysis

Among the continuous variables, normal distribution data were expressed as mean with standard deviation and skewed data were described as median with interquartile range. The Shapiro–Wilk normality test was used to test normality. Categorical variables were described as number of participants (percentage). Student’s t-test was used for normally distributed data and the Mann–Whitney U test was used for skewed data to identify the differences and compare clinical characteristics between groups. A restricted cubic spline curve was used for depicting the continuous relationships between the HRQOL scores and renal outcomes. Cox proportional hazard regression model was applied to survey the independent risk factors associated with CKD progression. The covariates for adjustment were selected considering our baseline data and other HRQOL-based studies of CKD. The proportional hazard assumption of the Cox proportional hazard model was tested using the Schoenfeld residuals test. Stratified Cox model was applied to the variables that violate the assumption of proportional hazard. HR and 95% CI were calculated to compare the risk of CKD progression. To access the mutual influence between variables, collinearity diagnosis was conducted. We performed propensity score matching analysis with the ‘MatchIt’ package in R. Propensity scores were estimated using logistic regression, and the nearest neighbor 1:1 matching was applied. Among the variables with a large difference in the baseline characteristics compared to the control group, we selected a variable that showed a statistically significant correlation with the renal outcome in the Cox proportional hazard model. The standardized difference (<0.1) was reviewed before and after matching to assess optimal matching. Data were analyzed using R (version 3.5.1; http://www.r-project.org). *P*-values < 0.05 were considered statistically significant.

## Supplementary information

Supplementary Figures

## Data Availability

Available as supplementary material when accepted.
