# Supplementary material for: Association between health related quality of life and progression of chronic kidney disease
Source: Sci Rep. 2019 Dec 20;9:19595. doi: 10.1038/s41598-019-56102-w (PMC6925203; doi:10.1038/s41598-019-56102-w)
Supplement: Supplementary file 1 — Supplementary Figures [file 41598_2019_56102_MOESM1_ESM.pdf]

Association between health related quality of life and progression of chronic kidney disease

Tae Ryom Oh, M.D.[1], Hong Sang Choi, M.D.[1], Chang Seong Kim, M.D., Ph.D.[1], Eun Hui Bae, M.D., Ph.D.[1], Yun Kyu Oh, M.D., Ph.D.[2], Yong-Soo Kim, M.D., Ph.D.[3], Kyu Hun Choi, M.D., Ph.D.[4], Soo Wan Kim, M.D., Ph.D.[1]* and Seong Kwon Ma, M.D., Ph.D.[1]*

[1]Department of Internal Medicine, Chonnam National University Medical School, Gwangju, Korea; [2]Department of Internal Medicine, Seoul National University College of Medicine, Seoul, Korea; [3]Department of Internal Medicine, The Catholic University of Korea, Seoul St. Mary's Hospital, Seoul, Korea; and [4]Department of Internal Medicine, College of Medicine, Institute of Kidney Disease Research, Yonsei University, Seoul, Korea;

Membership of the KNOW-CKD Investigator Group is provided in the Acknowledgments.

*These authors have contributed equally to this manuscript as correspondence authors.

Correspondence

*Soo Wan Kim, MD, PhD.

Department of Internal Medicine, Chonnam National University Medical School

42 Jebongro, Gwangju 61469, Korea

Tel: +82-62-220-6271, Fax: +82-62-225-8578

Email: skimw@chonnam.ac.kr

*Seong Kwon Ma, MD, PhD.

Department of Internal Medicine, Chonnam National University Medical School

42 Jebongro, Gwangju 61469, Korea

Tel: +82-62-220-6579, Fax: +82-62-225-8578

Email: drmsk@hanmail.net

Supplemental Material

Supplementary Figure S1. The score of each item composing physical component score by CKD progression

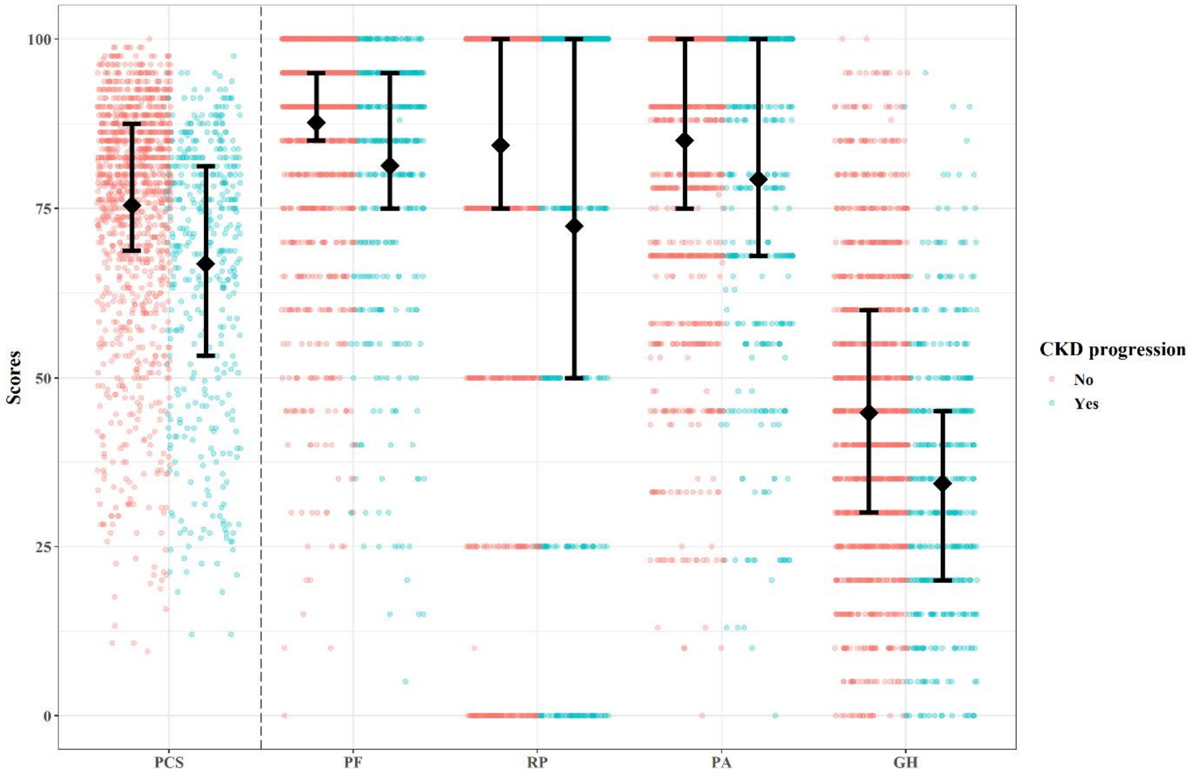

All items showed a significant difference between groups. Error-bar and square point mean interquartile range with an average of each item.

Abbreviations: PCS, physical component summary; PF, physical function; RP, role physical limitation; PA, bodily pain; GH, general health

Supplementary Figure S2. The score of each item composing mental component score by CKD progression

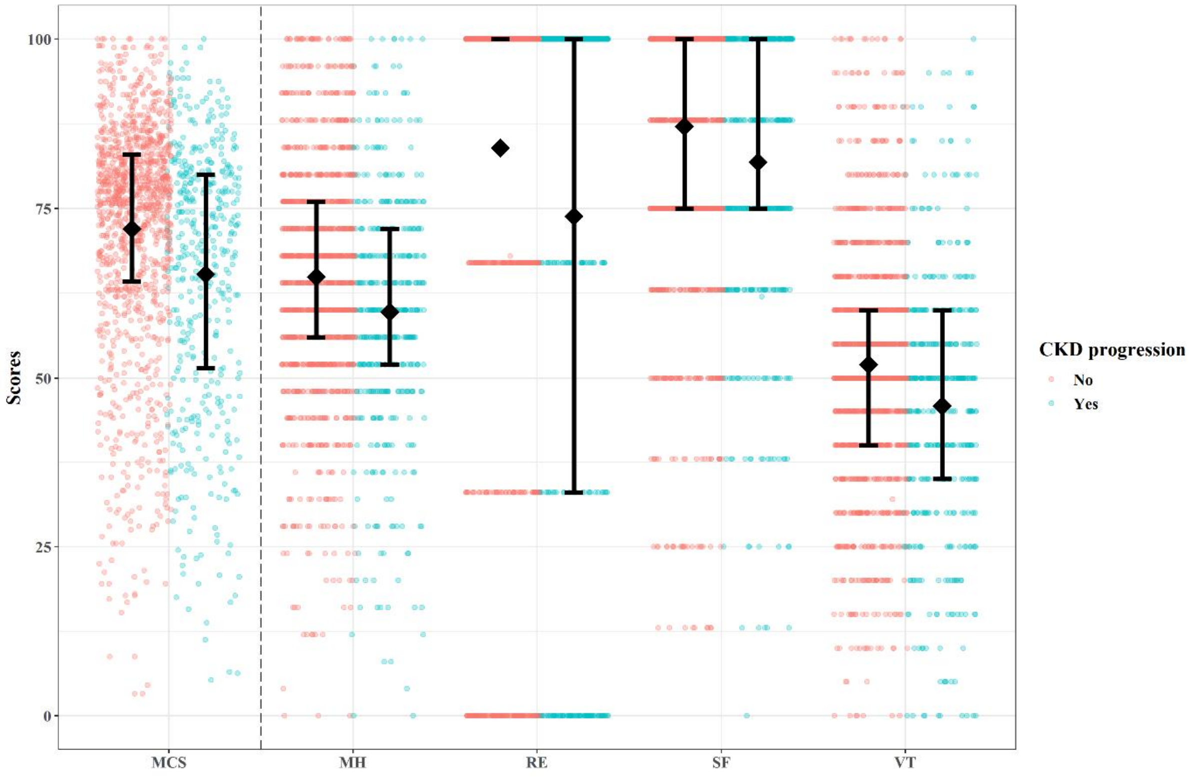

All items showed a significant difference between groups. Error-bar and diamond point mean interquartile range with an average of each item.

Abbreviations: MCS, mental component summary; MH, mental health, RE, role-emotional; SF, social function; VT, vitality

Supplementary Figure S3. Flow diagram of the study participants.

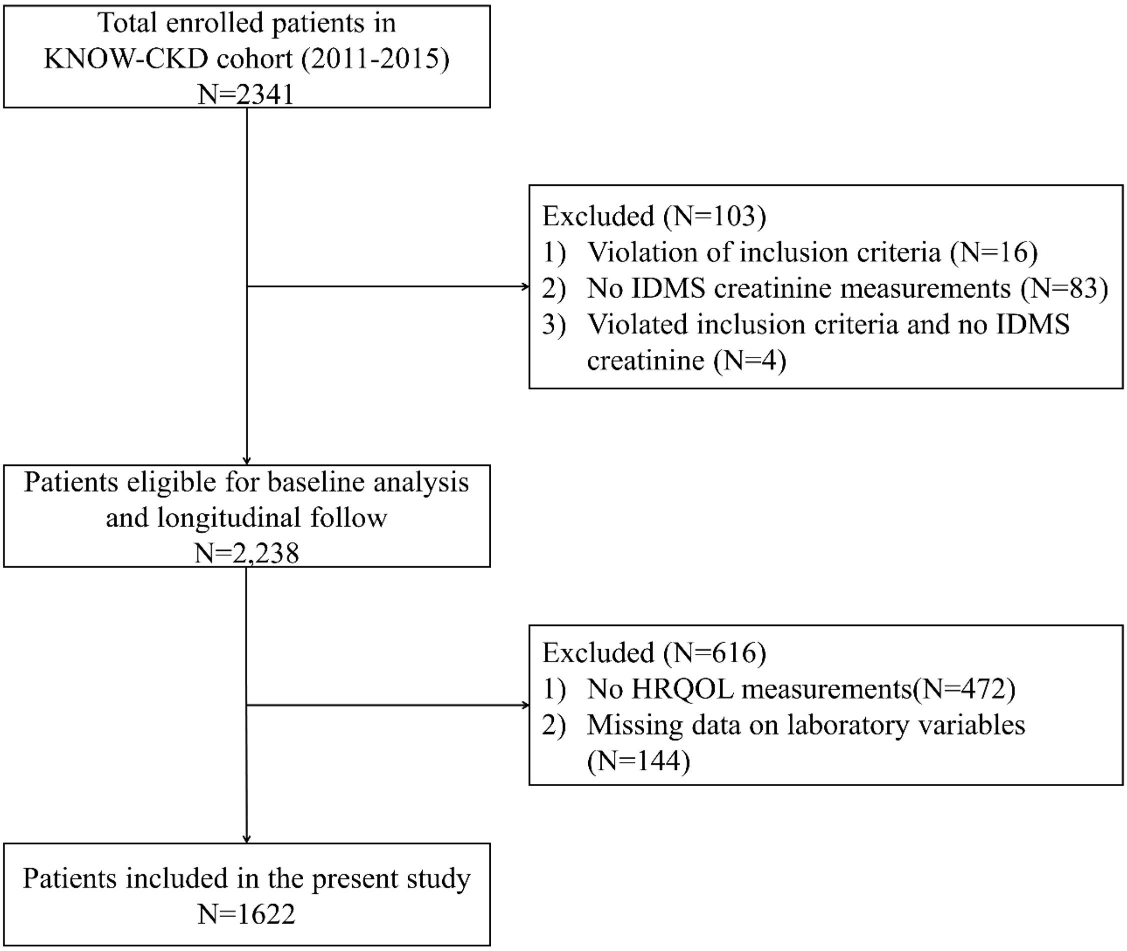

Abbreviations: Cr, creatinine; HRQOL, health related quality of life
